# Enhanced Photocatalytic and Antimicrobial Performance of Cuprous Oxide/Titania: The Effect of Titania Matrix

**DOI:** 10.3390/ma11112069

**Published:** 2018-10-23

**Authors:** Marcin Janczarek, Maya Endo, Dong Zhang, Kunlei Wang, Ewa Kowalska

**Affiliations:** 1 Institute for Catalysis, Hokkaido University, Sapporo 001-0021, Japan; m_endo@cat.hokudai.ac.jp (M.E.); zhang.d@cat.hokudai.ac.jp (D.Z.); kunlei@cat.hokudai.ac.jp (K.W.); kowalska@cat.hokudai.ac.jp (E.K.); 2 Department of Chemical Technology, Gdansk University of Technology, 80-233 Gdansk, Poland; 3 Institute of Chemical Technology and Engineering, Faculty of Chemical Technology, Poznan University of Technology, 60-965 Poznan, Poland

**Keywords:** photocatalysis, nanocomposites, heterojunction, Z-scheme, Cu_2_O, TiO_2_, antimicrobial properties

## Abstract

A simple, low-cost method was applied to prepare hybrid photocatalysts of copper (I) oxide/titania. Five different TiO_2_ powders were used to perform the study of the effect of titania matrix on the photocatalytic and antimicrobial properties of prepared nanocomposites. The photocatalytic efficiency of such a dual heterojunction system was tested in three reaction systems: ultraviolet-visible (UV-Vis)-induced methanol dehydrogenation and oxidation of acetic acid, and 2-propanol oxidation under visible light irradiation. In all the reaction systems considered, the crucial enhancement of photocatalytic activity in relation to corresponding bare titania was observed. The reaction mechanism for a specific reaction and the influence of titania matrix were discussed. Furthermore, antimicrobial (bactericidal and fungicidal) properties of Cu_2_O/TiO_2_ materials were analyzed. The antimicrobial activity was found under UV, visible and solar irradiation, and even for dark conditions. The origin of antimicrobial properties with emphasis on the role of titania matrix was also discussed.

## 1. Introduction

Titanium dioxide (TiO_2_, titania) is widely recognized as an efficient, stable and green photocatalytic material (long-term stability, chemical inertness, corrosion resistance and non-toxicity). Therefore, its application potential in photocatalysis is still growing and presently focused on areas such as environmental remediation (water treatment and air purification), renewable energy processes (i.e., water splitting for hydrogen production, conversion of CO_2_ to hydrocarbons), and self-cleaning surfaces [1,2,3]. However, one can distinguish two main problems in the wider application of TiO_2_. Firstly, the application of titania is still limited to the regions with a high intensity of solar radiation due to its wide bandgap (ca. 3.0 to 3.2 eV). Strategies such as titania doping [4], surface modification [5], semiconductor coupling [6], and dye sensitization [7] can be applied to incorporate visible light absorption to TiO_2_. Another important limitation decreasing photocatalytic activity is the recombination of the photogenerated electron-hole pairs caused by impurities, defects and other factors, which introduce bulk or surface imperfections into the titania crystal. The solution is the incorporation of species capable of promoting charge separation (e.g., TiO_2_ modification with metal ions, noble metals and heterojunction coupling with other semiconductors). Taking into consideration both described limitations, the methods to improve photocatalytic activity of TiO_2_ are similar. Therefore, a proper method selection to rectify both limitations is important to prepare a photocatalytic material with universal properties, active both in ultraviolet (UV) and visible light [8,9]. 

Copper (Cu) as a candidate for titania modification is a very promising material [10,11]. The first reason is the advantageous price and well-known antimicrobial properties. In comparison to other noble metals (gold, platinum and silver), recognized as very efficient co-catalysts of titania, copper, as the consequence of its abundance in the Earth’s crust, is an inexpensive material, 100 times and 6000 times cheaper than silver and gold, respectively. Copper can exist in the following oxidation states, i.e., Cu^0^, Cu^I^ and Cu^II^, and therefore the active copper species in TiO_2_ photocatalytic system can be recognized as copper oxides (Cu_2_O, CuO) and metallic copper. Copper oxidation states can also change as the consequence of sample drying [12,13,14] and reaction conditions [11,15]. For example, despite zero-valent copper being easily formed on the titania surface either by photodeposition [12,13,14] or radiolytic reduction [16], the contact with air results in fast oxidation of the copper, and the resultant photocatalysts possess different forms of co-existing copper species (mainly metallic core and oxide shell). It should be pointed that although copper is easily oxidized, stable zero-valent copper has been also reported when stabilized by titania aerogel [17]. Considering the above issues (the variety of copper forms and their relative instability), there is a difficulty in understanding their role in different reaction systems. Among copper oxides in relation to heterojunction with titania, Cu_2_O is one of the few p-type semiconductors which are inexpensive, non-toxic and widely available. The Cu_2_O/TiO_2_ p-n heterojunction system has promising application potential both in the oxidation of organic pollutants including very good antipathogenic properties [15,18,19,20,21,22,23,24,25,26,27,28,29,30,31,32] and in photocatalytic hydrogen production [11,29,33,34,35,36,37,38,39]. 

For example, Bessekhouad et al. proposed that under visible light irradiation, electrons from Cu_2_O were injected into the conduction band (CB) of TiO_2_, and at the titania surface could react with dissolved oxygen molecules inducing the formation of oxygen peroxide radicals (O_2_^∙−^) [21]. In the case of a UV system, an increase in the content of Cu_2_O resulted in enhanced efficiency, but resultant activities at high content of Cu_2_O were only slightly higher than that of pure TiO_2_ [21]. Similarly, Huang et al. found the improvement of photocatalytic activity for a Cu_2_O/TiO_2_ system induced by UV and visible light, i.e., 6 and 27 times higher photocatalytic activity than that for pure P25, respectively [30,31]. Moreover, it was found that an increase in the content of Cu_2_O resulted in higher photocatalytic activity (the highest activity for 70%-Cu_2_O content). A Cu_2_O/TiO_2_ p-n heterojunction system was also successfully applied for photocatalytic hydrogen generation. Zhang et al. prepared Cu_2_O/TiO_2_ composites through the deposition of copper on titania nanotube arrays [29]. Since the CB of Cu_2_O is more negative than that of TiO_2_, the excited electrons are quickly transferred from Cu_2_O nanoparticles to titania, leaving the holes on the valence band (VB) of Cu_2_O and leading to an effective reduction of protons to H2. 

Moreover, copper, especially copper (I) oxide, has been well known as a antimicrobial agent since ancient times. Due to its advantages, e.g., inexpensiveness, low toxicity and abundant sources, it has been applied to improve the photo-induced antimicrobial activity of titania. The proposed mechanisms include: (i) the structure of surface proteins are denaturated [40]; and (ii) the adsorbed copper ions induce oxidative stress in the bactericidal process [41], and the accumulation of copper ions inside bacteria [42]. It was found that the optimal balance of Cu_2_O and CuO content in Cu_x_O/TiO_2_ composite photocatalyst was important to achieve good antibacterial performance under visible light irradiation and dark conditions and, furthermore, Cu_2_O/TiO_2_ was reported to be more active than CuO/TiO_2_ and CuNPs/TiO_2_ [18].

To the authors’ best knowledge there is still no comprehensive research paper considering the effect of a titania matrix in Cu_2_O-titania heterojunction system for photocatalytic and antimicrobial properties. The clarification of this issue is important in order to analyze application perspectives of such photocatalysts. In this study, to evaluate the role of titania matrix, five types of titania were considered. Heterojunctions between Cu_2_O and different TiO_2_ were prepared as physical mixtures of powders. Enhanced photocatalytic properties were discussed based on three reaction systems: methanol dehydrogenation and oxidation of acetic acid under UV/vis irradiation, and 2-propanol oxidation under vis irradiation. The antimicrobial properties of prepared composite photocatalysts were tested by using *E. coli* and *C. albicans*.

## 2. Materials and Methods 

### 2.1. Preparation of Cu_2_O/TiO_2_ Photocatalysts

TiO_2_ samples, selected for the preparation procedure, were supplied by several sources: P25 (AEROXIDE^®^ TiO_2_ P25, Nippon Aerosil, Yokkaichi, Japan), ST-01 (ST-01, Ishihara Sangyo, Yokkaichi, Japan) ST-41 (ST-41, Ishihara Sangyo, Yokkaichi, Japan), TIO-6 (TIO-6, Catalysis Society of Japan, Tokyo, Japan), RUT (rutile nanopowder, Sigma-Aldrich, Saint Louis, MO, USA). Cu_2_O was supplied by Wako Pure Chemicals, Tokyo, Japan. All materials were used as received, without further processing. Cu_2_O/TiO_2_ composites were prepared by physical mixing of Cu_2_O and TiO_2_ powders in an agate mortar. Titania samples were mixed with different contents of Cu_2_O resulting in preparation of the composites containing 1, 5, 10 and 50 wt % of Cu_2_O. The time of grinding (5 min) was the same for all samples to ensure appropriate homogeneity of prepared composite powders. 

### 2.2. Characterization

The ultraviolet-visible (UV-Vis) diffuse reflectance spectra (DRS, JASCO, Tokyo, Japan) were recorded on JASCO V-670 equipped with PIN-757 integrating sphere using BaSO_4_ as a reference. Gas-adsorption measurements of prepared titania samples were performed on a Yuasa Ionics Autosorb 6AG surface area and pore size analyzer, Osaka, Japan. Specific surface area (SSA) was calculated from nitrogen adsorption at 77 K using the Brunauer–Emmett–Teller equation. X-ray diffraction (XRD) patterns were collected using an X-ray diffractometer (Rigaku intelligent XRD SmartLab with a Cu target, Tokyo, Japan).

### 2.3. Photocatalytic Activity Tests

The photocatalytic activity of prepared photocatalysts was tested in three reaction systems: (1) decomposition of acetic acid under UV/vis irradiation, (2) dehydrogenation of methanol under UV/vis irradiation, and (3) oxidation of 2-propanol under vis irradiation (λ > 420 nm: Xe lamp, water IR filter, cold mirror and cut-off filter Y45). For activity testing, 50 mg of photocatalyst was suspended in 5 mL of aqueous solution of (1) methanol (50 vol %), (2) acetic acid (5 vol %), and (3) 2-propanol (5 vol %). The methanol dehydrogenation system was also tested in the presence of platinum (samples: Pt/TiO_2_, Pt/Cu_2_O/TiO_2_): hydrogen hexachloroplatinate(IV) (H_2_PtCl_6_·6H_2_O) was added for adjustment to 2 wt % loading on photocatalyst powders. The suspension for reaction (2) was bubbled with argon before irradiation. The 35-mL testing tubes were sealed with rubber septa, continuously stirred and irradiated in a thermostated water bath. Amounts of liberated (1) carbon dioxide in gas phase, (2) hydrogen in gas phase, and (3) acetone in liquid phase (after powder separation) were determined by GC-TCD (1-2) (Shimadzu GC-8A equipped with a thermal conductivity detector, Shimadzu Corp., Kyoto, Japan) and GC-FID (3) (Shimadzu GC-14B equipped with a flame ionization detector, Shimadzu Corp., Kyoto, Japan).

### 2.4. Antimicrobial Activity Tests with Xenon Lamp Irradiation

Cu_2_O/TiO_2_ samples, bare titania and cuprous oxide (ca. 7.1 g/L) were dispersed in *Escherichia coli* K12 (ATCC29425) or *Candida albicans* (isolated from patients (throat smear) with immunodeficiency disorders that cause candidiasis (collection from West Pomeranian University of Technology, Szczecin, Poland)) suspension at concentrations of ca. 1–5 × 10^8^ cells/mL (*E. coli* K12) or 1–5 × 10^4^ cells/mL (*C. albicans*) in a test tube with stirring bar, and then irradiated with xenon lamp (with cold mirror (CM2) and UV-D36B filter; 300 < λ < 420 nm or CM1 and Y-45 filter; λ > 420 nm) or kept in the dark. As a control, bacterial or fungal suspension without titania was also tested. Serial dilutions (10^−1^–10^−6^) of microbial suspension were prepared and aliquots of suspensions were inoculated on Plate Count Agar (Becton Dickinson Company, Franklin Lakes, NJ, USA for *E. coli* K12) or Malt Extract Agar (Merck Millipore Corporation, Burlington, MA, USA for *C. albicans*) media at 0, 0.5, 1, 2 and 3 h. Media were incubated at 37 °C overnight, colonies were counted, and the colony-forming unit was determined.

### 2.5. Antibacterial Activity Test with Solar Irradiation

Cu_2_O/TiO_2_ (ST-01) or bare titania (ST-01) (0.5 g/L), was dispersed in *E. coli* K12 (ATCC29425) suspension at a concentration of ca. 1 × 10^8^ cells/mL in a glass container with stirring bar, and circulated through glass tubes and irradiated under solar radiation (Sapporo, sunny day, June 2018) or kept in the dark. As a control, bacterial suspension without titania was also tested. The later experimental procedure was the same as that described above. 

## 3. Results and Discussion

### 3.1. Characterization of Cu_2_O/TiO_2_ Samples

**Figure 1:**
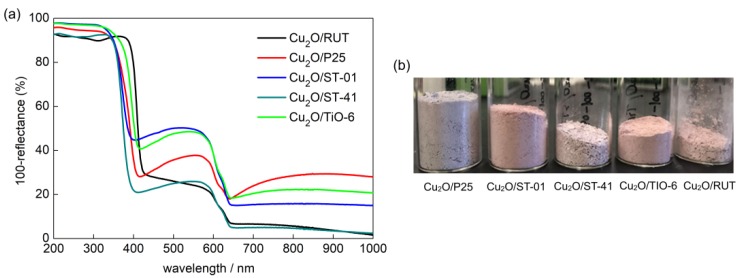
Diffuse reflectance spectra of Cu_2_O (5 wt %)/TiO_2_ samples (**a**) with respective photographs (**b**).

**Figure 2:**
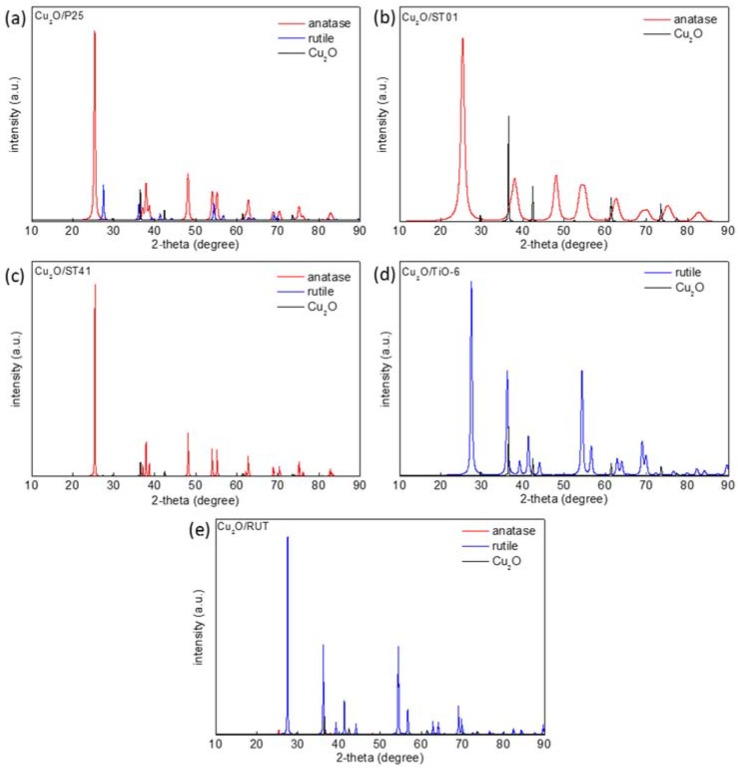
X-ray diffraction (XRD) diffractograms of Cu_2_O-modified titania samples: (**a**) Cu_2_O/P25, (**b**) Cu_2_O/ST-01, (**c**) Cu_2_O/ST-41, (**d**) Cu_2_O/TIO-6, (**e**) Cu_2_O/RUT.

**Figure 3:**
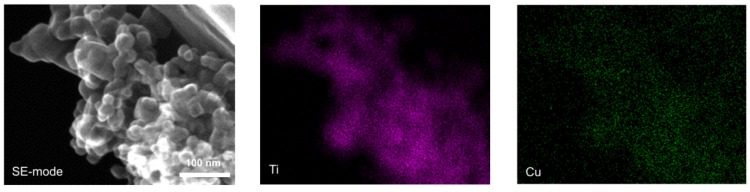
Scanning transmission electron microscopy (STEM) images with energy dispersive spectroscopy (EDS) mapping of Cu_2_O/P25 sample. Mapping colors: Ti-violet; Cu-green.

Five commercially available TiO_2_ were selected to perform the study. Table 1 shows the main properties of the samples, which represent diversified types of titania matrix, e.g., different morphology, phase content, crystallite size and specific surface area. Figure 1a shows diffuse reflectance spectra of prepared Cu_2_O/TiO_2_ with 5 wt %-content of cuprous oxide. The strong absorption at UV range is due to bandgap excitation of titania, and a narrower bandgap of rutile than that of anatase clearly correlates with the absorption edge at longer wavelengths. The absorption peak between 500–600 nm is characteristic of the presence of Cu_2_O. Although the content of Cu_2_O in each sample was approximately the same, one can observe significant differences in the shape of Cu_2_O-absorption region between samples containing a different titania matrix. It is possible to observe the dependency between the size of TiO_2_ particles and the height of Cu_2_O-originated absorption peak. The Cu_2_O/TiO_2_ samples with small-particulate titania (ST-01 and TIO-6) are characterized by the strongest 500–600 nm absorption peak of Cu_2_O. The crystallite size of Cu_2_O determined by XRD analysis was 65 nm and BET surface area: 23 m^2^·g^−1^. The prevalence of anatase or rutile in a titania matrix did not influence the Cu_2_O-originated absorption peak. Another confirmation for the phase presence (anatase, rutile, Cu_2_O) in the prepared samples are the XRD results, shown in Figure 2. Cuprous oxide was confirmed in all samples (black patterns after subtraction of titania patterns). Moreover, titania peaks did not change after grinding, which proves that physical mixing was delicate without changing crystal properties (It is known that strong grinding/milling could destroyed titania crystals.). Figure 3 shows scanning transmission electron microscopy (STEM) images with energy dispersive spectroscopy (EDS) mapping, which indicate that Cu_2_O particles are uniformly distributed on the surface of titania. 

### 3.2. Photocatalytic Activity

#### 3.2.1. Ultraviolet-Visible (UV-Vis)-Induced Methanol Dehydrogenation

During the reaction of methanol dehydrogenation (H_2_ system) under deaerated conditions in the presence of titania and copper oxides the following reactions may be considered:

TiO_2_ + hν (λ > 290 nm) → e^−^ + h^+^, (generation of electrons and holes) (1a)

Cu_2_O + hν (λ > 290 nm) → e^−^ + h^+^, (1b)

CH_3_OH + h^+^ → HCHO + H^+^, (2)

Cu^+^ + e^−^ → Cu, (3)

H^+^ + e^−^ → 0.5 H_2_, (4)

**Figure 4:**
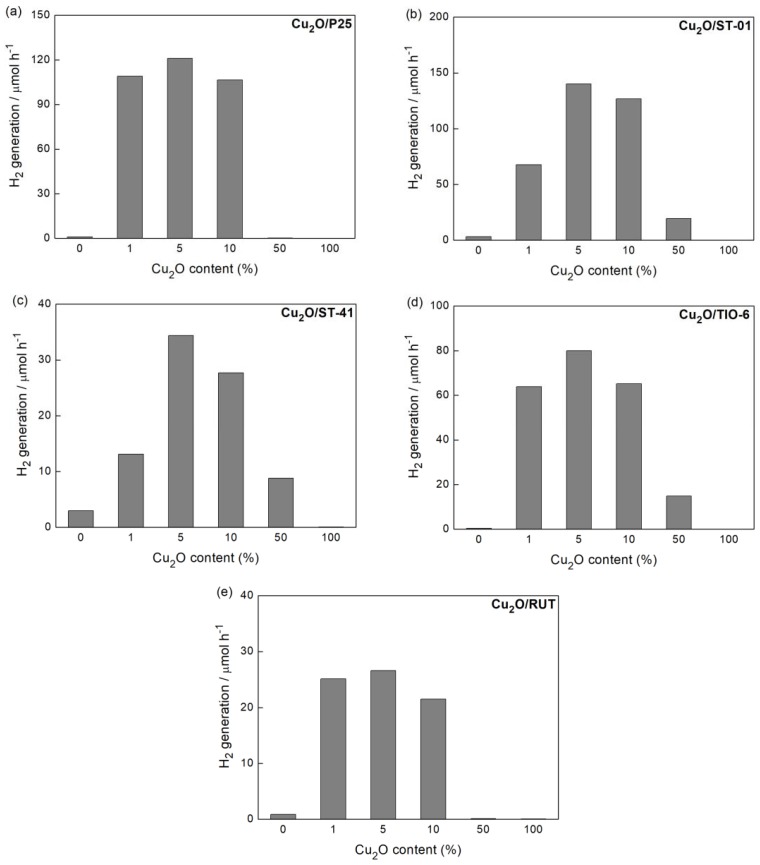
Ultraviolet-visible (UV-Vis) photocatalytic activity of samples: (**a**) Cu_2_O/P25, (**b**) Cu_2_O/ST-01, (**c**) Cu_2_O/ST-41, (**d**) Cu_2_O/TIO-6, (**e**) Cu_2_O/RUT, prepared with corresponding Cu_2_O content in methanol dehydrogenation.

At the beginning of irradiation, copper oxide is reduced by photoexcited electrons, resulting in the formation of copper deposits on the surface of the photocatalyst (3). In this system, methanol plays the role of a hole scavenger (2), as presented in Figure 4.

**Figure 5:**
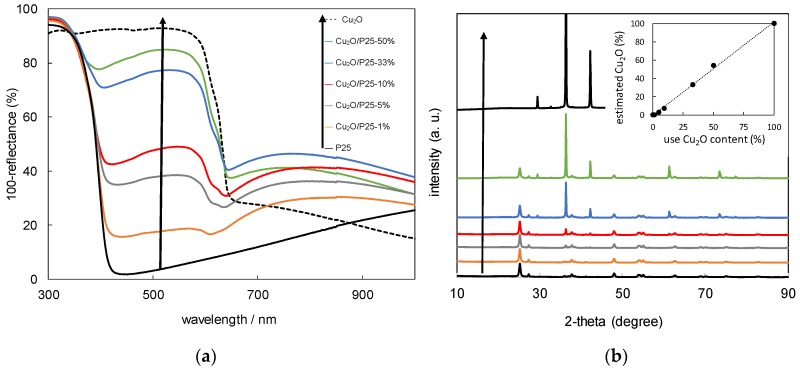
(**a**) Diffuse reflectance spectra and (**b**) XRD diffractograms of Cu_2_O/P25 samples with different Cu_2_O content; Inset: Correlation between use and estimated (XRD) content of Cu_2_O in the samples.

As a primary issue, the best copper content (wt %) for Cu_2_O/TiO_2_ system corresponding to highest photocatalytic activity was determined for further studies. The following copper contents were considered: 1%, 5%, 10% and 50%. For all titania-cuprous oxide heterojunctions, the highest photocatalytic activity was observed for 5 wt % of Cu_2_O (Figure 4). By increasing the Cu_2_O content, the hydrogen evolution rate decreased because of the increase of charge recombination effect [11] or inner filter effect (competition for photons between two semiconductors). The high content of Cu_2_O (50 wt %) caused a significant decrease of photocatalytic activity, probably due to the increase in the opacity and light scattering (shielding effect) influencing photon absorption (irradiation passing through the photocatalyst suspension) [43]. Indeed, characterization of Cu_2_O/P25 samples with different content of Cu_2_O clearly showed a significant increase in light absorption with an increase in cuprous oxide content at vis range (Figure 5a). Additional XRD analysis confirmed the presence of both titania and cuprous oxide in all hybrid samples (Figure 5b), and the estimated content of cuprous oxide in hybrid materials was almost the same as that used for the preparation of samples (inset in Figure 5b). 

**Figure 6:**
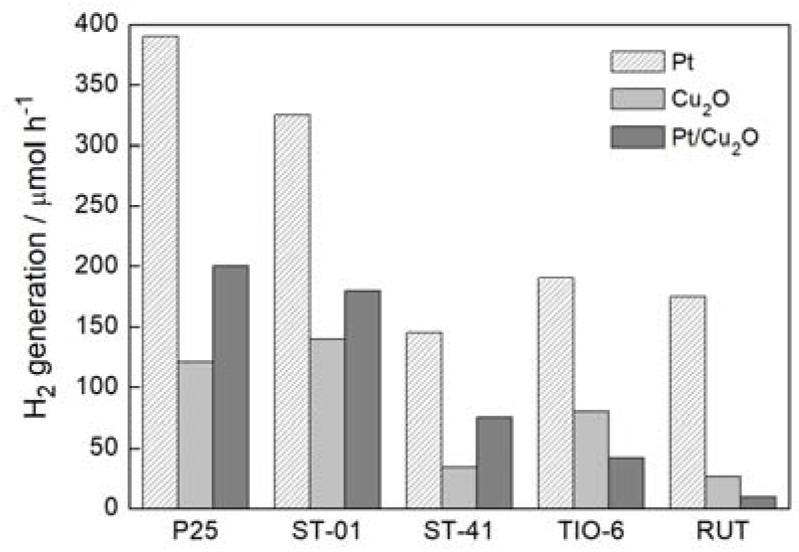
Comparison of UV/Vis photocatalytic efficiency of Cu_2_O/TiO_2_, Pt/Cu_2_O/TiO_2_ and Pt/TiO_2_ samples in methanol dehydrogenation. TiO_2_: P25, ST-01, ST-41, TIO-6, RUT.

Figure 6 shows the photocatalytic activity in hydrogen system for Cu_2_O/TiO_2_ with different titania matrix. These activities were compared with corresponding samples: Pt/TiO_2_ and Pt/Cu_2_O/TiO_2_. Obviously, the highest photocatalytic activities were obtained for Pt/TiO_2_, due to higher work function (6.35 eV vs. 4.7 eV) and smaller overvoltage for hydrogen evolution for platinum than that for copper. Although, the activity of Cu_2_O/TiO_2_ was 2–3 times lower than that for Pt/TiO_2_, it should be remembered that copper is much cheaper than platinum. Moreover, bare titania is practically inactive in this system (Figure 6), and thus evolution of hydrogen on this cheap photocatalyst is quite promising. Additionally, it should be pointed out that another possible mechanism, i.e., type II heterojunction (transfer of photo-generated electrons from CB of Cu_2_O to CB of TiO_2_ with opposite transfer of photo-generated holes) could be rejected due to the inactivity of bare titania (Figure 6). In contrast to the conclusions of Dozzi et al. [44], the synergistic effect of Pt-Cu was not observed in this reaction system. This is not surprising since Pt-modified titania is one of the most active photocatalysts for hydrogen evolution, and thus formation of other charge carriers’ transfers (not only from CB of titania to Pt), e.g., to VB of Cu_2_O, should result in hindering of the overall activity. Photocatalytic activities of Pt/Cu_2_O/TiO_2_ were slightly higher than that of Cu_2_O/TiO_2_ excluding samples based on rutile (TIO-6 and RUT). The higher activity for co-modified anatase samples than that for Cu_2_O/TiO_2_ could originate from an increase in the content of active sites for hydrogen evolution (both on copper and platinum deposits), whereas the reason for the lowest activity for co-modified rutile samples is unclear. It is possible that more negative CB of rutile than that of anatase (as recently reported [45,46]) could result in two types of co-existing mechanisms (Z-scheme and type II heterojunction), i.e., (1) for Pt-modified titania, photoexcited electrons from CB of Cu_2_O migrates to CB of titania (type II heterojunction) and then to Pt deposits (together with directly excited electrons from VB of titania); (2) for Cu_2_O/TiO_2_ system, Z-scheme mechanism should be preferential (due to inactivity of bare titania); (3) for Pt-modified Cu_2_O/TiO_2_, similar levels of CBs position for rutile and cuprous oxide could result in the circulation of photogenerated electrons between both semiconductors, instead of their transfer to noble metals’ deposits, i.e., VB(Cu_2_O) → CB(Cu_2_O) → CB(rutile) → VB(Cu_2_O). It should be reminded that Pt was deposited in situ, and thus it is highly possible that it could be randomly deposited either on cuprous oxide or on titania. The formation of bimetallic deposits Cu-Pt with metal segregation is also possible, as already reported for titania photocatalysts modified with Au-Cu [47], and Ag-Cu [13,16]. To clarify the mechanism, detailed studies on sample characterization, and reference experiments for pre-modified titania with platinum are presently under study.

**Figure 7:**
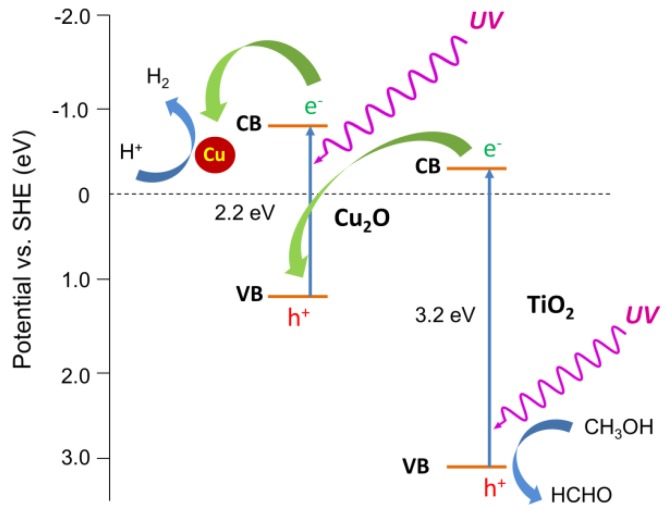
Two-step charge separation system (Z-scheme) with Cu-Cu_2_O Schottky barrier as the mechanism for UV/Vis-induced methanol dehydrogenation with Cu (formed in-situ)/Cu_2_O/TiO_2_ photocatalysts.

UV/Vis photocatalytic activity of Cu_2_O/TiO_2_ hybrid photocatalysts in this reaction system is enhanced by the combination of the synergistic effect of formed metallic copper and Cu_2_O caused by the effect of Schottky barrier created between zero-valent copper and cuprous oxide (hindering charge carriers’ recombination in cuprous oxide—its main shortcoming) [33,48] with a Z-scheme system (see Figure 7) as a type of mechanism of photogenerated carriers migration to form an efficient two-step charge separation system. Moreover, it should be pointed out that the proposed Cu-Cu_2_O-TiO_2_ nanostructure limits the problem of Cu_2_O instability, i.e., self-oxidation by photo-generated holes (recombine with CB electrons from titania) and self-reduction by photo-generated electrons (which migrate to zero-valent copper).

#### 3.2.2. UV/Vis-Induced Acetic Acid Oxidation

**Figure 8:**
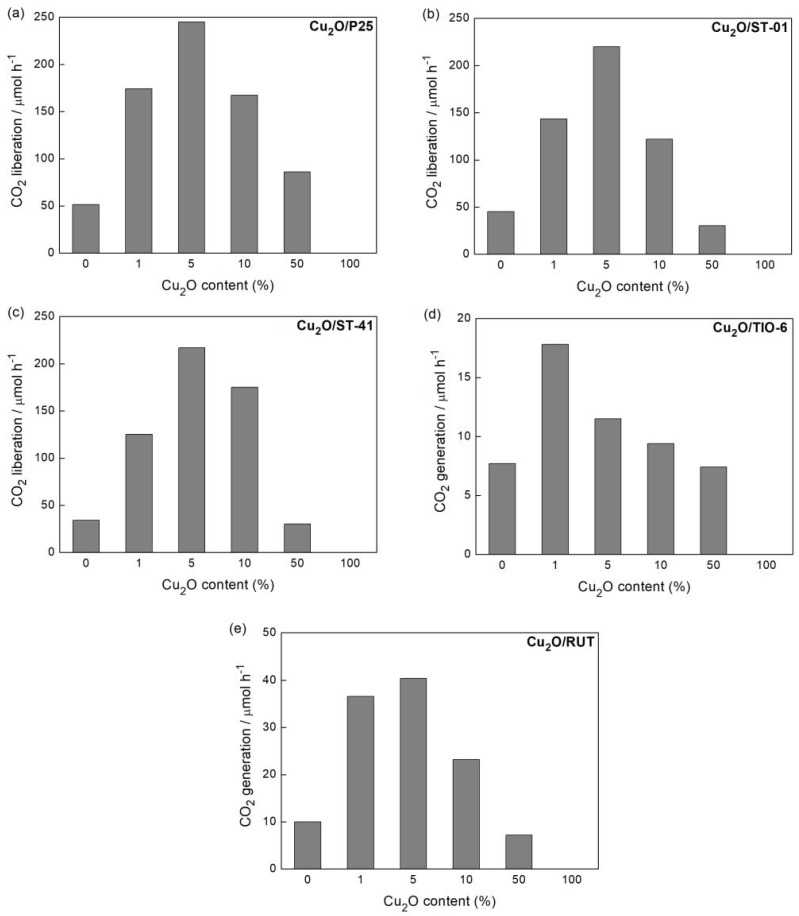
UV/Vis photocatalytic activity of samples: (**a**) Cu_2_O/P25, (**b**) Cu_2_O/ST-01, (**c**) Cu_2_O/ST-41, (**d**) Cu_2_O/TIO-6, (**e**) Cu_2_O/RUT, prepared with corresponding Cu_2_O content in acetic acid oxidation.

**Figure 9:**
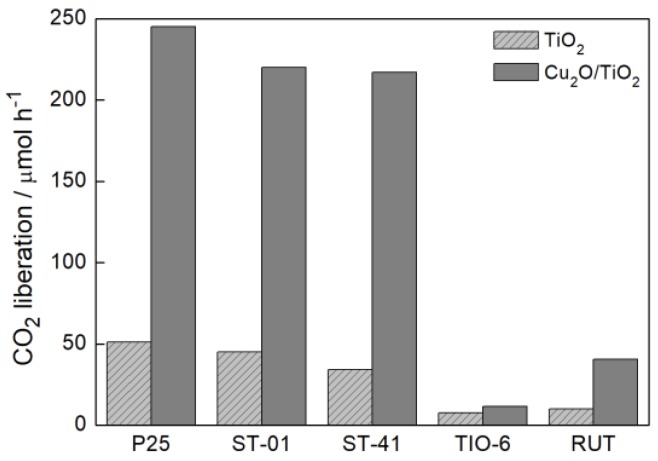
UV/Vis photocatalytic efficiency of Cu_2_O/TiO_2_ samples with different titania matrix and corresponding bare TiO_2_ in acetic acid oxidation.

As in the previous reaction system, photocatalytic activity in the UV/Vis-induced acetic acid oxidation increased with an increase in Cu_2_O content until 5 wt % (Figure 8). The exception was only for the TIO-6 sample, where the maximum of activity was found for 1 wt % of Cu_2_O. A further increase of Cu_2_O content was detrimental for the photocatalytic activity. Pure Cu_2_O was almost inactive in this reaction because of a high recombination rate [49]. Therefore, either its high content in this hybrid photocatalyst or dark color (inner filter and shielding effects as discussed in Section 3.2.1) caused low photocatalytic efficiency under UV/vis irradiation. It should be pointed out that fine titania (ST-01) and mixed-phase titania (P25) are well known as highly active samples for this reaction (it is difficult to find more active titania photocatalysts, and probably only decahedral anatase particles (faceted anatase with two kinds of facets: eight {001} facets and two {001} facets) exhibited slightly higher activity than that of P25 [50]); and thus an increase in their activities by ca. 4–5 times by modification with small content of Cu_2_O (Figure 9) is highly promising for other oxidation reactions and even the complete mineralization of organic pollutants. 

**Figure 10:**
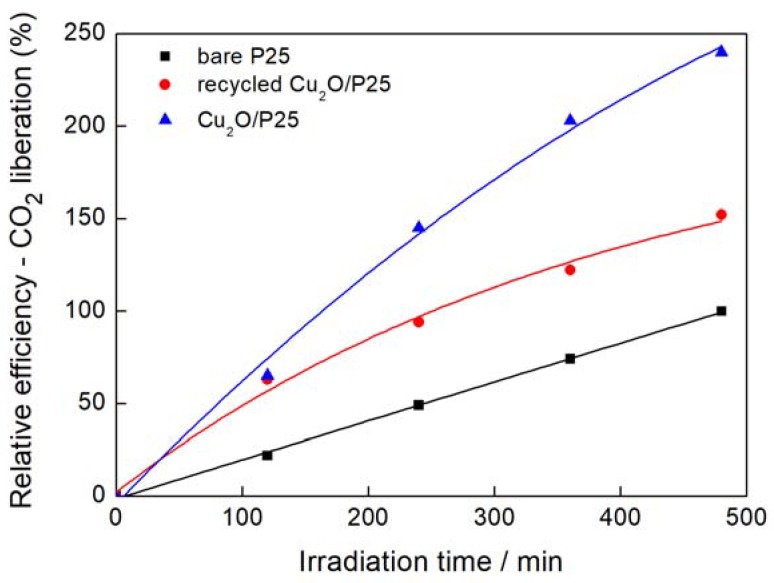
UV/Vis photocatalytic activity of Cu_2_O/P25—the photostability study considering the reusability of Cu_2_O/P25.

For Cu_2_O/TiO_2_ samples with anatase as a dominant titania phase, the significant improvement of photocatalytic activity was achieved (Figure 9). The reaction efficiency was ca. 4–5 higher than that for corresponding bare TiO_2_ regardless of particle size of titania, and catalytic activity (in the absence of irradiation) of Cu_2_O/TiO_2_ samples was negligible. It is important to mention that the preparation of these samples by physical mixing is not detrimental for overall photocatalytic performance of obtained heterojunction systems, which is really high. Significantly smaller improvement was observed only for rutile-based hybrid photocatalysts, in particular, for TIO-6. Figure 10 shows the results of the long photoactivity experiment for a Cu_2_O/P25 sample considering the reusability of the photocatalyst and the comparison to the activity of bare P25. After 6 h of irradiation, an almost linear course of CO_2_ liberation was still observed suggesting good photostability in this reaction system during continuous irradiation (the close reaction system with possible equilibrium between different forms of copper). However, the loss of photocatalytic activity of the recycled sample (losing the linear course) was observed for 2 h of irradiation. Fortunately, continued irradiation (2–6 h) resulted in stable photocatalytic activity. It was confirmed (by XRD analysis) that the content of the Cu_2_O in recycled sample decreased, with simultaneous appearance of CuO and Cu (0) in comparison to fresh Cu_2_O/P25 sample. Therefore, to extend the reusability of prepared samples, additional operations to strengthen the connection between these two components, e.g., annealing, or preparation of advanced nanostructures should be considered. For example, a core (Cu_2_O)/shell (titania) nanostructure will be investigated in our future study, similarly to the reported Cu_2_O/Au nanostructure with gold nanoparticles (NPs) deposited on Cu_2_O nanowires [51].

**Figure 11:**
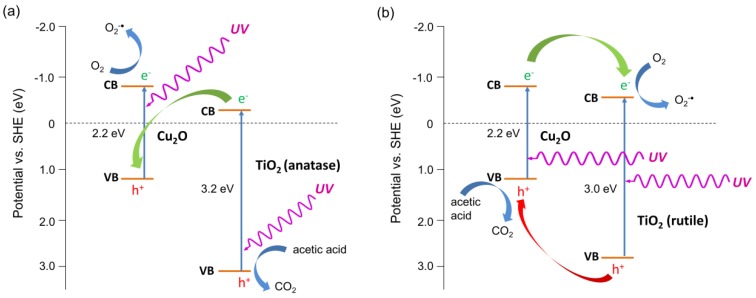
Proposed mechanisms of UV/Vis-induced acetic acid oxidation with Cu_2_O/TiO_2_ photocatalysts: Z-scheme for anatase samples (**a**) and p-n heterojunction (type II) for rutile sample (**b**).

Two types of mechanism could be considered, similarly to H_2_ system, i.e., Z-scheme and p-n heterojunction (type II), as shown in Figure 11. Under UV light irradiation, both Cu_2_O and TiO_2_ could be excited, and either photo-generated electrons in TiO_2_ could recombine with photo-generated holes in the VB of Cu_2_O or electrons in Cu_2_O, and holes in TiO_2_ could migrate to the CB of TiO_2_ and VB of Cu_2_O, respectively. The first mechanism seems to be preferential resulting in the generation of charges with stronger redox potential (more negative electrons and more positive holes). It is thought that photocatalytic activity for the oxidation reactions depends directly on the oxidation potential of holes, as recently reported for an oxygen activation study by M. Buchalska et al. [45]. The same study by M. Buchalska et al. proved that anatase was a stronger oxidant than rutile, due to the more positive position of the VB. Therefore, lower activities of rutile samples could be easily explained by less positive potential of the VB than that in anatase titania. Consequently, more negative potential of the CB in the rutile case may result in higher probability of type II heterojunction than Z-scheme, and thus not so high improvement of photocatalytic activity. Although, heterojunction II results in lower redox potential than the Z-scheme, the transfer process described above is thermodynamic favorable, and may result in the prolongation of the lifetime of excited electrons and holes, inducing higher quantum efficiency. Acetic acid is decomposed either by oxidative species such as O_2_^∙−^ and OH^∙^, formed by the reaction of generated electrons with dissolved oxygen and by the reaction of generated holes from VB of TiO_2_ with water, or directly by positive holes. It must be remembered that the lack of holes’ consumption can be the reason for Cu_2_O photocorrosion [52], and thus the proposed Z-scheme for anatase samples should be responsible for both high activity (strong redox ability) and stability. 

#### 3.2.3. Visible Light Photocatalytic Activity 

**Figure 12:**
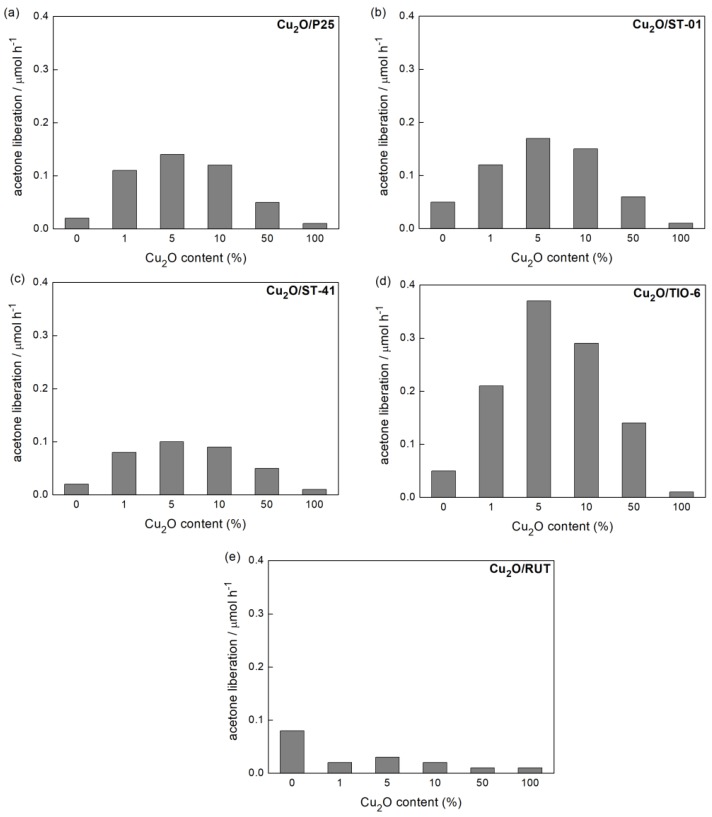
Visible light-photocatalytic activity of samples: (**a**) Cu_2_O/P25, (**b**) Cu_2_O/ST-01, (**c**) Cu_2_O/ST-41, (**d**) Cu_2_O/TIO-6, (**e**) Cu_2_O/RUT, prepared with corresponding Cu_2_O content in 2-propanol oxidation.

**Figure 13:**
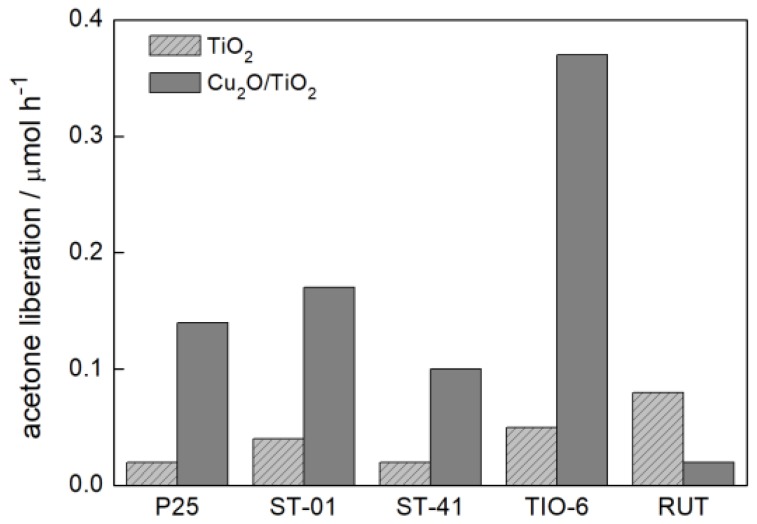
Visible light-photocatalytic efficiency of Cu_2_O/TiO_2_ samples with different titania matrix and corresponding bare TiO_2_ in 2-propanol oxidation.

**Figure 14:**
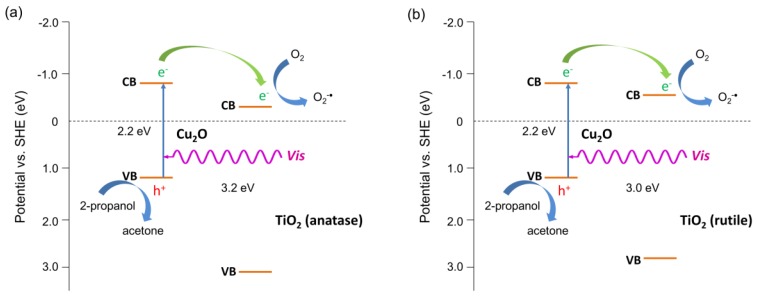
Schematic mechanism of heterojunction (type II) for vis-induced 2-propanol oxidation with Cu_2_O/TiO_2_ photocatalysts for anatase (**a**) and rutile (**b**).

Cu_2_O as a 2.2 eV-band gap energy-semiconductor absorbs visible light. Therefore, one can expect that a hybrid system of Cu_2_O with titania should be more active in the visible light than Cu_2_O and titania alone. The results of photocatalytic activity for vis-induced 2-propanol oxidation (Figure 12 and Figure 13) confirmed this expectation. Figure 14 shows the scheme of heterojunction system (Cu_2_O/TiO_2_) with visible light-activation of Cu_2_O. The visible light-induced electron transfer between CB of Cu_2_O and CB of titania should play the key role in the photocatalytic efficiency of this system. Similarly, as in the case of previous reaction systems 5 wt %-Cu_2_O content resulted in the highest activity of Cu_2_O/TiO_2_ photocatalysts, independently of the titania matrix (Figure 12a–d), but with the exception of Cu_2_O/RUT, where vis photocatalytic activity of bare RUT was higher than that in hybrid system (Figure 12e). The highest improvement of photocatalytic activity in relation to bare titania (ca. 6 times) was found for rutile sample: Cu_2_O/TIO-6 (Figure 12d).

The crucial question is why the vis-induced photocatalytic properties of samples Cu_2_O/TIO-6 and Cu_2_O/RUT are so different. Densities of lattice defects (DEF; also known as electron traps (ETs)) equivalent to the concentration of Ti^3+^ were estimated for different types of titania in the earlier study [53]. The values of DEF were 50, 84, 38, 242 and 18 μmol·g^−1^ for P25, ST-01, ST-41, TIO-6 and RUT, respectively. The higher DEF of titania matrix favors the higher vis light-photocatalytic activity in the considered reaction system (Cu_2_O/TIO-6), but the lowest DEF of RUT corresponds to no reaction rate improvement. The presence of Ti^3+^ ions can be important for the efficiency of the visible light-induced reaction on Cu_2_O/TiO_2_. Photogenerated electrons from Cu_2_O can be captured by Ti^4+^ ions and thus, being reduced to Ti^3+^. Ti^3+^ ions (with prolonged lifetime), participate in electron trapping resulting in retarded charge recombination. These observations are with agreement with the concept of Xiong et al. on the role of Ti^3+^ ions in Cu_2_O/TiO_2_ heterojunction system [23]. Moreover, the significant difference in enhancement factor between anatase and rutile hybrid samples of similar vis activity before modification (ST-01 and TIO-6, due to high content of DEF) could indicate that localization of CB of titania in respect to that of cuprous oxide is crucial. Therefore, higher proximity between cuprous oxide and rutile than that between cuprous oxide and anatase could facilitate an electron migration. Moreover, as M. Buchalska et al. suggested [45] the lower redox potential of the excited electron of rutile than that of anatase resulted in more efficient O_2_^∙^^−^ generation, and thus higher activity in reactions involving photo-excited electrons as the main mechanism pathway. Similarly, a higher activity of rutile than anatase was found for plasmonic photocatalysis by gold-modified titania, in which “hot” electron transfer from plasmonic gold NPs to CB of titania was proposed [54]. 

### 3.3. Antimicrobial Properties of Cu_2_O/TiO_2_

**Figure 15:**
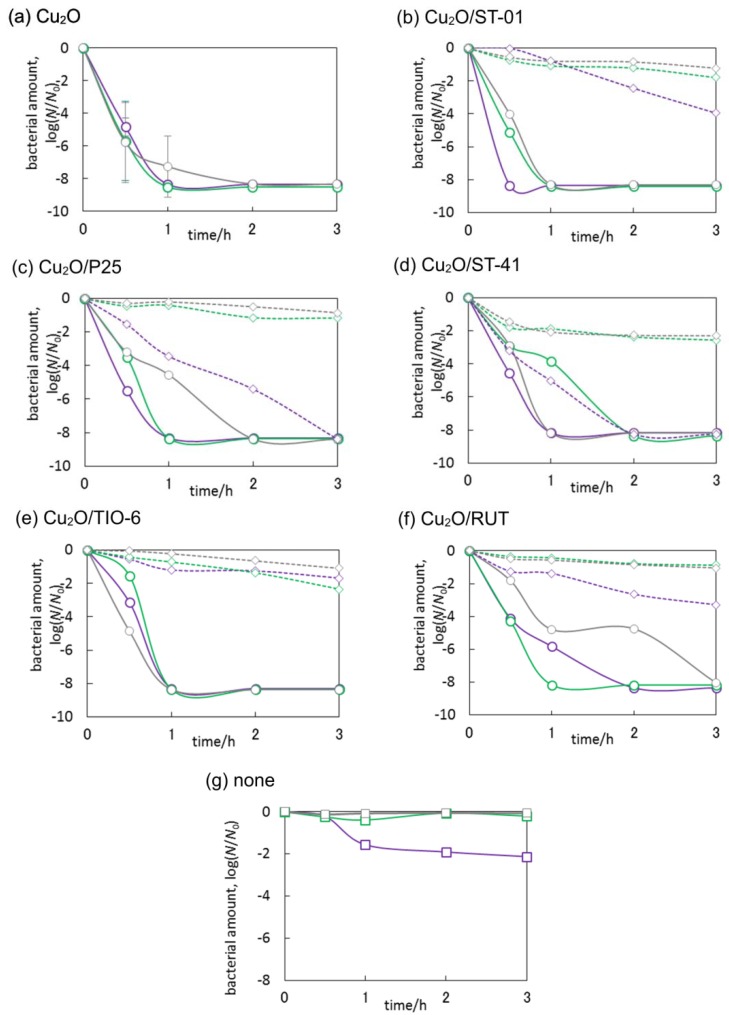
*E. coli* K12 survival shown as CFU/mL during inactivation of bacterial cells; (**a**–**f**) in the dark (grey symbols), under UV irradiation (300 < λ < 420 nm; violet symbols) and under vis irradiation (λ > 420 nm; green symbols) on bare (diamond, dashed line) and modified titania (circle, solid line), (**g**) *E. coli* K12 survival without titania in the dark, under UV and visible. Error bars (Cu (I) oxide) indicate the standard deviations calculated from two or three independent measurements.

At first, the bactericidal property of Cu_2_O was investigated in the dark, under UV and visible light, and the results obtained are shown in Figure 15a. Cuprous oxide exhibited high bactericidal activity, and irradiation with UV and visible light slightly enhanced the intrinsic activity of Cu (I). It means that light irradiation could promote electron transfer between Cu and bacterial cells (Cu extracts electrons from bacteria, causing proteins denaturation [40]) and generation of reactive oxygen species (ROS) resulting in the cell’s inactivation. It should be pointed out that the contact between Cu (I) and bacteria is essential for bacterial inactivation, in addition, surface Cu-ions are crucial for bactericidal property [55].

Bare titania exhibited bactericidal property under UV light irradiation, due to the generation of ROS, such as ^•^OH, O_2_^−•^ and H_2_O_2_, in which the activity seemed to arise from the particle size. In contrast, compared to the activity of anatase and without titania, bare rutile titania did not show significant enhancement under UV irradiation, which is not surprising because the photocatalytic activity of rutile is generally lower than that of anatase [54], as already discussed for acetic acid oxidation.

Under UV light irradiation, all Cu-modified anatase samples (Cu_2_O/ST-01, Cu_2_O/P25 and Cu_2_O/ST-41) showed the enhancement of bactericidal activity. Qiu et al. have already reported that the bactericidal activity under visible light irradiation attributed to multi-electron reduction by electrons on Cu (II) in Cu_x_O clusters which was transferred from the VB of titania by inter-facial charge transfer (IFCT), in contrast, Cu (I) in Cu_x_O clusters showed anti-pathogen effect in the dark [55]. In this regard, it is proposed that similar mechanism could be responsible for enhanced UV-activity of Cu_2_O/TiO_2_ photocatalysts, i.e., IFCT from titania to Cu, as well as hindering of charge carriers’ recombination (as discussed above). Interestingly, the dark activity of Cu_2_O/ST-41 was higher than the visible one. It is probable that the contact between Cu (I) and bacteria could be affected, i.e., large titania ST-41 (crystal size = ca. 70 nm) could not cover Cu particles; on the other hand, small titania particles of ST-01 and P25 (ca. 8 and 21 nm, respectively) could cover NPs of cuprous oxide inhibiting the direct contact with bacteria and/or release of Cu ions from the surface of Cu (I). In the contrary, Cu (I)-modified rutile titania did not show the highest activity under UV irradiation, furthermore, the tendency of activity was different between two rutile samples (Cu_2_O/TIO-6 and Cu_2_O/RUT). In the case of Cu_2_O/TIO-6, it is probable that direct bactericidal activity of Cu (I) in the dark might exceed the generation of ROS which was attributed to the activity of the multi-electron reduction on Cu by IFCT under UV and visible light irradiation, and vice versa in the case of Cu_2_O/RUT. 

**Figure 16:**
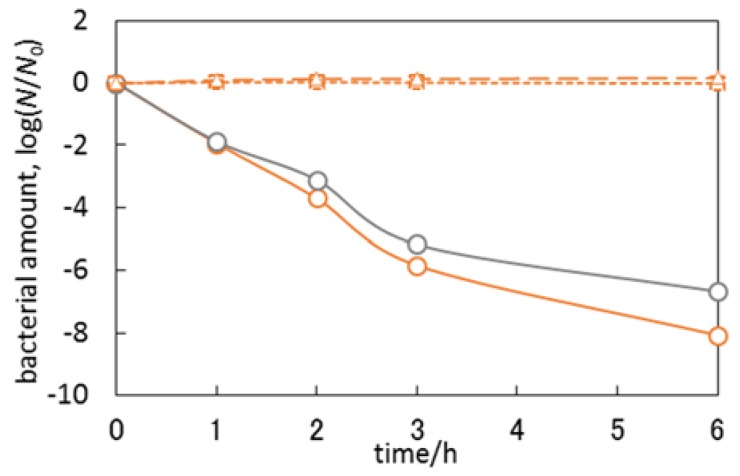
Bactericidal activities of Cu_2_O/ST-01 (circle, solid line), bare titania ST-01 (square, dashed line) and reference experiments without any photocatalyst (triangle, dashed line) under solar light irradiation (orange) and in the dark (gray).

The most active photocatalyst, i.e., Cu_2_O/ST-01 was additionally tested under natural solar radiation, and data obtained are shown in Figure 14. Interestingly, no bactericidal activity was observed both in the absence of photocatalyst and for bare titania (ST-01), despite the fact that titania ST-01 showed some activity under UV irradiation (Figure 15b,g), possibly because the light intensity of solar radiation was quite low compared to an artificial source of light (xenon lamp), used in laboratory experiments (Figure 16). On the other hand, Cu_2_O/ST-01 showed high activity under solar light and better than that in the dark (ca. one order of magnitude). It is thought that additionally to dark activity of Cu (I), and similarly to acetic acid oxidation, enhanced generation of reactive oxygen species could result in activity improvement (Z-scheme shown in Figure 11a). 

Concluding, it is clear that the bactericidal property of Cu_2_O/TiO_2_ originates mainly from the presence of cuprous ions, and photocatalytic activity only slightly enhanced the effect. Therefore, it is suggested that positively charged Cu (I) could both attract a negatively charged bacterial membrane (due to the presence of lipopolysaccharide) and inactivate cells by intrinsic activity of Cu (I). 

**Figure 17:**
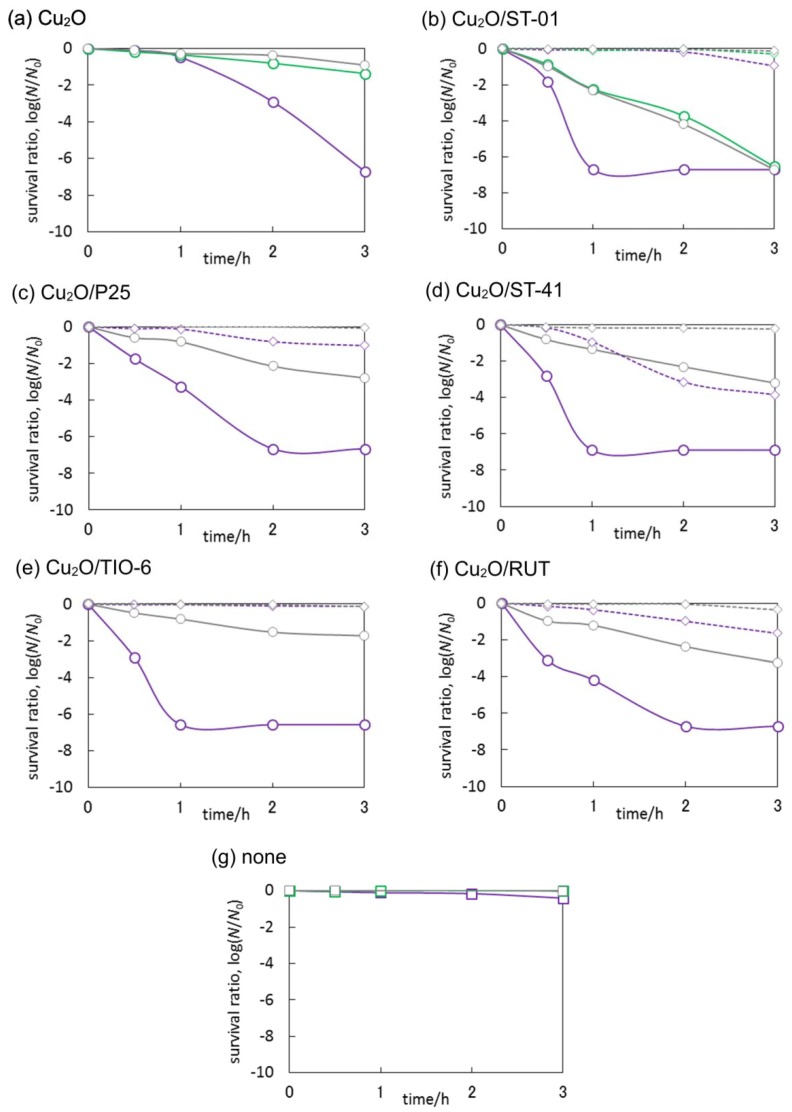
*C. albicans* survival shown as CFU/mL during inactivation of fungal cells; (**a**–**f**) in the dark (grey symbols), under UV irradiation (300 < λ < 420 nm; violet symbols) and under vis irradiation (λ > 420 nm; green symbols) on bare (diamond, dashed line) and modified titania (circle, solid line), (**g**) *E. coli* K12 survival without titania in the dark, under UV and visible.

In order to investigate deeper the antimicrobial effects of cuprous oxide/titania system, the fungicidal activities (*C. albicans*) were additionally studied, and data obtained are shown in Figure 17. It was found that copper (I) oxide remarkably suppressed fungal survival only under irradiation with UV light. The initial fungicidal rate was quite slow, and then accelerated after 1 h of irradiation. It is important to take into account the structure of fungal cells (yeast) and the surface charge of the cell wall. Although the electrostatic potential of *C. albicans* cells’ surface is negative [56], their cell walls are rigid, they have a nuclear membrane and the size of cell is larger than that of bacteria. Therefore, despite Cu (I) oxide being easily in contact with cells, it could take a longer time to kill fungal cells than bacterial cells. It could be considered that the fungicidal mechanisms are similar to bacterial ones, and in addition, as reported by K. Danmek et al., Cu inhibits the activity of cellulase (in *Aspergillus melleus*), which could induce the inhibition of glycan decomposition and eventually the lack of nutrients [57]. The inactivation of *C. albicans* by cuprous oxide/titania was greatly promoted by UV light irradiation, and the activities in the dark were not so effective, unlike bactericidal activity in the dark. All Cu_2_O-modified titania samples under UV irradiation reached detection limit within 1–2 h. Therefore, it is proposed that, in the case of fungi, although the influence of intrinsic activity of Cu (I) is slow, the effect of ROS on cell components might be fast, resulting in the difference of velocities between Cu_2_O and Cu_2_O/TiO_2_. The activities under visible light (Cu (I) oxide and Cu_2_O/ST-01) were almost the same as that in the dark suggesting that enhancement of antifungal activity was not only caused by possible formation of superoxide anion radicals (Figure 14). Accordingly, it is proposed that significant enhancement of activity under UV irradiation for Cu_2_O/TiO_2_ photocatalyst could result from a Z-scheme mechanism (Figure 11a) leading to either enhanced generation of hydroxyl radicals (by both holes from VB of titania and electrons from CB of cuprous oxide) or direct decomposition of fungal cells by photogenerated charge carriers. It will be clarified in our further studies by comparing ROS generated in UV, visible and dark conditions. Summarizing, it was found that fungicidal activities of cuprous oxide in the dark were promoted by modifying with titania, indicating that the activity was not derived from the sole activity of Cu_2_O but also by heterojunction of Cu_2_O and TiO_2_. 

## 4. Conclusions

In summary, a simple and low cost-method, realized by mixing of copper (I) oxide and titania, yields an efficient hybrid photocatalyst. By using a different titania matrix, one can adjust the both photocatalytic and antimicrobial properties of the resultant material. Considering the methanol dehydrogenation reaction, the enhanced efficiency of Cu_2_O/TiO_2_ photocatalysts originates from the combination of the Cu-Cu_2_O Schottky barrier with a Z-scheme system. A large improvement of photocatalytic activity of copper (I) oxide-titania system in comparison to corresponding bare titanium(IV) oxides was found for UV/Vis-induced acetic acid oxidation, mainly for a titania matrix with anatase as a dominant phase. Taking into consideration oxidative reactions, Cu_2_O/anatase is an example of a very efficient Z-scheme system, induced by UV/Vis irradiation, with a good perspective of application for solar systems dedicated for wastewater treatment, confirmed by good photostability, but the reusability of prepared photocatalysts needs further improvement. The mechanism of photocatalytic activity of rutile-based samples could be described as the type II heterojunction system. Furthermore, these two mechanistic variants, Z-scheme and heterojunction-type II, were also suggested for visible light-induced oxidation of 2-propanol for anatase and rutile-based samples, respectively. The photocatalytic efficiency in this system was correlated with the concentration of Ti^3+^ ions in a titania matrix (density of lattice defects)—the highest concentration of Ti^3+^ for TIO-6 means the highest vis-photocatalytic activity rate. Another important issue examined in this study was the antimicrobial property of Cu_2_O/TiO_2_ materials. All prepared samples possessed bactericidal and fungicidal properties, which were observed for UV, visible, solar irradiation, and even for dark conditions. It was concluded that antimicrobial activity depends not only on intrinsic properties of Cu_2_O but also heterojunction between copper (I) oxide and titania.

## Figures and Tables

**Table 1:** TiO_2_ samples selected for the study.

Sample Name	Anatase * (%)	Rutile * (%)	Crystallite Size/nm		Specific Surface Area/m^2^·g^−1^
			Anatase	Rutile	
P25	83.8	16.2	21	37	59
ST-01	100	-	8	-	298
ST-41	98.3	1.7	70	124	11
TIO-6	-	100	-	16	105
RUT	1.7	98.3	55	82	4

* Crystalline composition without consideration of amorphous phase.
